# Autobiographical Case Report of Experiences With Pleomorphic Xanthoastrocytoma

**DOI:** 10.7759/cureus.19668

**Published:** 2021-11-17

**Authors:** Cherry Liu, Nutan Winston

**Affiliations:** 1 Anesthesiology, Riverside Community Hospital, Riverside, USA

**Keywords:** plemorphic, levetiracetam, seizure activity, brain tumors cns tumors, xanthoastrocytoma

## Abstract

Pleomorphic xanthoastrocytoma is a rare brain tumor of WHO grade II designation. This case report describes the author's experience with the discovery of the tumor, living with the tumor, and eventual treatment and aftermath of how it affected her life and her understanding of the medical system.

## Introduction

Pleomorphic xanthoastrocytoma (PXA) is a type of astrocytoma that is very rare and accounts for <1% of all astrocytomas [1]. The most common presenting symptoms are seizures [1]. This case report describes the author’s experience with discovery, management, and eventual surgery removing the PXA from the left ventricle of her brain. Studies have shown that the prognosis of PXA is favorable, with three to five-year survival rates of greater than 80% and greater than 75%, respectively, with optimal management guided by case reports and case series due to the rarity of the tumor [1].

## Case presentation

Diagnosis

I was diagnosed with a brain tumor in my left ventricle incidentally in 2014 at the age of 20, after volunteering to do brain magnetic resonance imaging (MRI) in college for a professor studying Alzheimer’s disease. My only symptom was consistent “déjà vu” moments occurring about three to four times daily. At the time, my professor quickly got me an appointment with a neurologist who accepted the school insurance and I had a more thorough MRI done. The resulting images showed what neurologists assumed was a meningioma that was about 2.0 x 2.1 x 2.2 cm^3 in volume in my left ventricle presumably pressing on my hippocampus, explaining those symptoms. They referred me to get an electroencephalogram (EEG), where I was officially diagnosed with seizure activity. I was prescribed levetiracetam 500 mg twice a day (BID), which decreased these events to about one to two times daily. The dosage was increased to 750 mg BID and the symptoms were well controlled as long as I was compliant with the medication.

Over the next two years, I continued to take levetiracetam without any change in symptoms and continued to get an MRI every six months to monitor the status of the tumor. I also established care with a neurosurgeon to be prepared for any advances in symptoms or changes in the status of the tumor. During my gap year between graduating and applying to medical school, I started developing additional symptoms that resembled absence seizures that my neurologist labeled as complex partial seizures associated with amnesia based on my description of the experiences, despite increasing my levetiracetam dosage to 1000 mg BID.

Surgery

Thus, in the winter of 2017, in between my medical school interviews, I had a left temporal-parietal craniotomy for the left posterior hippocampal brain tumor. The final pathology showed a pleomorphic xanthoastrocytoma of WHO grade II with BRAF v600E mutation.

Pre-surgery Preparation

I was blessed to have a very comprehensive team of providers to discuss my case and provide me care. My professor, neurologist, and neurosurgeon set up a meeting with the neurologist and neurosurgeon at the facility that I ultimately chose to pursue the surgery at. I had to have another MRI and an ophthalmological exam as well prior to the surgery. My surgeon was also very understanding of my worries about having this surgery done right before one of my medical school interviews. He planned the surgery strategically during a two-month gap between my scheduled interviews at the time.

Post-operative Complications

Post-operatively, I have a full tonic-clonic seizure including tongue biting that resulted in a full day of neuro ICU stay due to post-operative inflammation from the surgery. Additionally, I had some minor difficulty with reading certain words, spelling, and verbalizing my thoughts coherently. The levetiracetam dose was increased to 1500 mg BID post-operatively. Despite these small issues, I had a relatively unremarkable stay and a smooth recovery. I was discharged on post-operative day two after the drain was removed with six days of enoxaparin and levetiracetam decreased to 1000 mg BID.

Follow-up

Over the next years, I continue to have MRI and follow up every six months including during medical school. After the seizure, I was deemed unfit to drive for two years by my neurologist, but my classmates and medical school were very supportive in assisting me with accommodations. I continued to slowly decrease my levetiracetam dosage with my neurologist over the course of the next four years. Currently, I am not taking any medication for seizures and have been seizure-free for five years. I still have the scar from the craniotomy, and the only irritation it gives me is flaking skin in dry environments.

## Discussion

**Figure 1:**
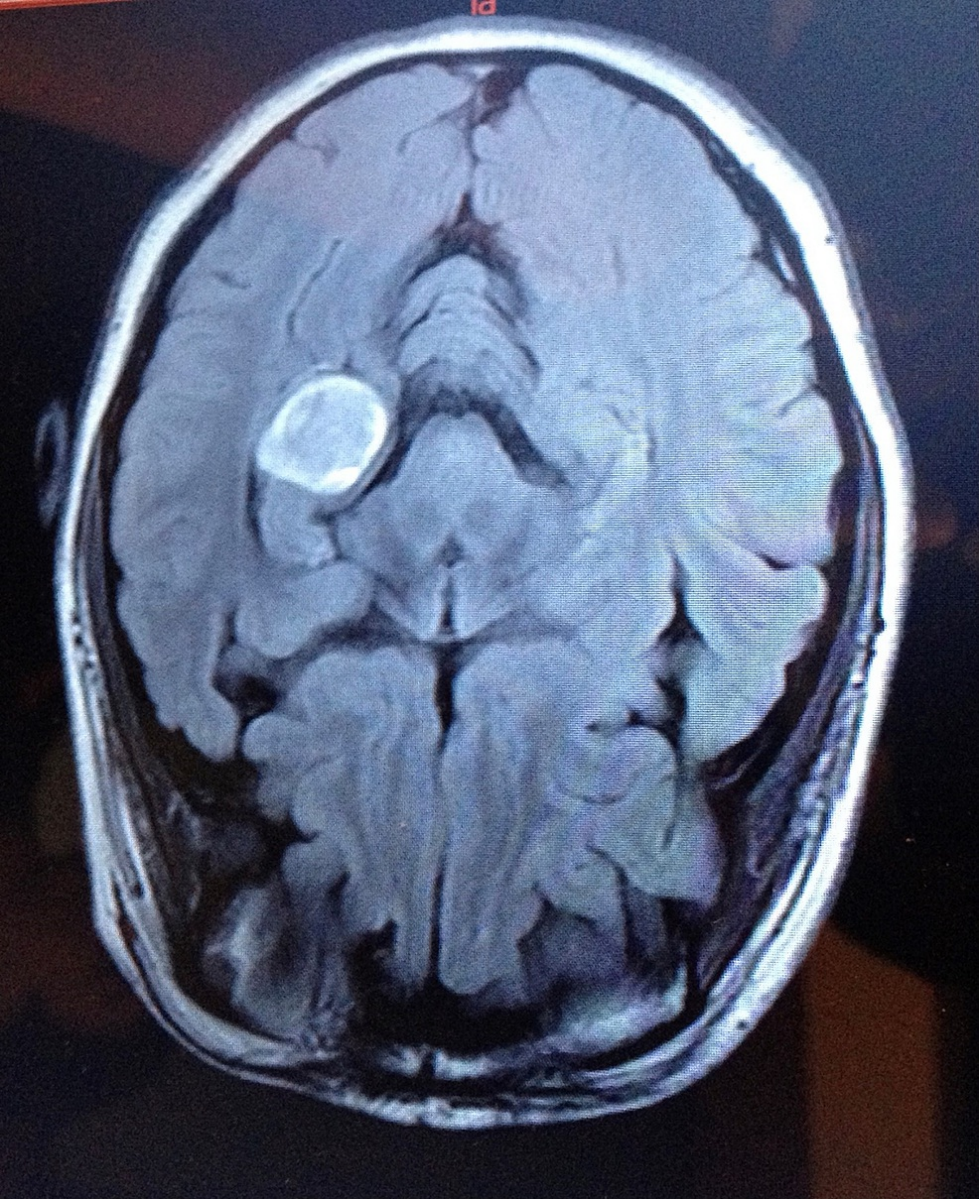
MRI image of my pleomorphic xanthoastrocytoma, showing an enhancing lesion with a small cystic component.

PXA is a rare WHO grade II CNS tumor of astrocytic origin with a favorable prognosis, especially if resected [1-3]. In fact, PXA is so rare that it accounts for only <1% of all astrocytomas [1]. The most common presenting symptom is a seizure, with other symptoms including increased intracranial pressure, headaches, nausea, vomiting, diplopia, or somnolence [1]. The mean age of diagnosis is 29 ± 16 years [1]. In a study back in 1999 consisting of 35 females and 36 males aged 26 ± 16 years, researchers found that 98% of the tumors were supratentorial and 49% were in the temporal lobe, similar to that of my tumor [3]. Interestingly, initially due to its position in the left ventricle, my neurologists and neurosurgeons assumed that the tumor was a meningioma. It was only after resection and pathology that the diagnosis of a PXA was able to be correctly made. However, with images provided from my MRI scans, it can be seen that my tumor did fit the gross description of a PXA, as it is an enhancing lesion with a small cystic component (Figure 1) [1].

While the majority of PXA tumors have a favorable diagnosis, there has been a recent addition to the 2016 World Health Organization classification of tumors in 2016 involving anaplastic pleomorphic xanthoastrocytoma (A-PXA) as grade III glial tumors [4]. The study of many case presentations has found that the most commonly mutated gene in PXAs is BRAF V600E, with over 2/3 cases having this mutation [1]. This gene is an intracellular component of the mitogen-activated protein kinase (MAPK) pathway and was the same mutation as the tumor that was extracted from my left ventricle [2]. Those with this mutation have a more favorable prognosis compared to those who have what is considered the wild-type mutation [1,2,4]. Those with a positive TERT promoter mutation had a higher incidence of having A-PXA [4].

The overall survival rate of those with PXA is very favorable, with those that have a BRAF mutation having a better prognosis than those without [1]. The five-year survival with PXA is greater than 75%, but A-PXA has a less favorable outcome [1]. The best management for PXA is complete surgical correction, which was what I successfully underwent with my care team. [1]. Other treatments such as radiotherapy are reserved for recurrent or residual disease, and systemic therapies are not as useful for treatment for this type of tumor [1]. Trials utilizing therapies such as BRAF inhibitor (dabrafenib) in combination with MEK inhibitor (trametinib) are still being studied but have encountered difficulty in clinical investigations due to the rarity of PXA [1].

Additional learning points

As a fairly independent first child of first-generation immigrants who did not speak much English, my first instinct after that meeting with my professor was immediately “What do I do next?” In the following months, I learned more about how to navigate the medical system from the patient side more than I learned in even my third year of medical school. From being put on a two-month waitlist to see a neurologist until my professor made some calls for me, to calling my insurance company (thankfully I was still under 26 and covered by my parent’s company insurance) to see which primary care providers, MRI facilities, and neurosurgeons were considered “in-network,” I was able to understand the frustration and confusion many of my patients currently feel today. Since I am the only person in my family with any sort of even a science background, I needed to translate my diagnosis to my parents while also struggling to understand what was happening myself.

The moment of the diagnosis also took away some of my independence, now that I could no longer drive for an indefinite amount of time, and forced me to rely on others in a way that I was definitely extremely uncomfortable with. Similarly, I was extremely worried about the financial burden my diagnosis would cause my parents. It was then that I learned about and was very thankful for the “pre-existing conditions” clause that would require insurance companies to cover my condition even when my father changed companies for work. Ultimately, when I saw the final sticker cost of my surgery and the short stint in the ICU, I was extremely grateful to be in a situation where I was under 26 years old and still covered under my parents’ insurance. If I had not gotten that MRI for the Alzheimer’s study and thus gotten diagnosed much later due to my initial extremely nonspecific symptoms, I may not have been so lucky!

It was also through this experience that I was able to be more vulnerable to others. As a young person, I used to think I was invincible. Yet, with a single diagnosis, I was suddenly rendered unable to drive. Ultimately, the main impact of this tumor was the limitations it placed on my driving ability. While I was in college and during my gap year, I lived in an area with robust public transportation where driving a vehicle was unnecessary and even possibly a nuisance. However, my first two years of medical school were only possible with the supportive administration and my classmates because it was in an area where having a car was almost an absolute necessity for transportation. Thankfully, I lived with classmates and the administration assigned them similar schedules to mine. By the third year, these same friends basically gave me the driving lessons I so desperately needed due to my four-year hiatus from behind the wheel. Thus, when I was cleared to drive, I was finally able to regain my independence and start my rotations that were located throughout the state.

## Conclusions

A brain tumor is a life-changing diagnosis that is often devastating. I was lucky enough to have not only an incredibly incidental find before any obvious symptoms, but also a supportive care team, family, classmates, and school administration who supported me through not only the diagnosis but its treatment and the aftermath. While a PXA is a WHO grade II CNS tumor, it is a relatively benign tumor with a five-year survival rate of over 75%. The best treatment for PXA is complete resection, but those with A-PXA may need additional radiotherapy, systemic therapies, or other novel treatment strategies.
